# Supplementary material for: Virus specificity and nucleoporin requirements for MX2 activity are affected by GTPase function and capsid-CypA interactions
Source: PLoS Pathog. 2024 Mar 21;20(3):e1011830. doi: 10.1371/journal.ppat.1011830 (PMC10986937; doi:10.1371/journal.ppat.1011830)
Supplement: S1 Table — Oligonucleotides used for cloning and genomic DNA amplification. (PDF) [file ppat.1011830.s001.pdf]

S1 Table. PCR primers

MX2 mutants	
Mx2-SfiI-F	CTCTGGCCGAGAGGGCCATGTCTAAGGCCCACAAGCCTTG
Mx2-myc-SfiI-R	CTCTGGCCAGAGAGGCCTCACAGATCCTCTTCAGAGATGAGTTTCTGCTCGTGGATCTCTTTGCTGG AGAA
Mx2-SfiI-R	CTCTGGCCAGAGAGGCCTCAGTGGATCTCTTTGCTGGAG
Mx2-M574D-F	TGATCCAACTTCAGTTCAGAGACGAGCAGATGGTTTTTTGTC
Mx2-M574-R	GACAAAAAACCATCTGCTCGTCTCTGAACTGAAGTTGGATCA
Mx2-Y651D-F	CCAGATCCCATTTATAATTCAGGATTTTATGCTCCGAGAGAATGGTGACTCC
Mx2-Y651D-R	GGAGTCACCATTCTCTCGGAGCATAAAATCCTGAATTATAAATGGGATCTGG
Mx2-YRGK-AAAA F	GTTGAAAAATATGAAAAGCAGGCTGCAGCCGCGGAGCTTCTGGGATTTGTCAAC
Mx2-YRGK-AAAA R	GTTGACAAATCCCAGAAGCTCCGCGGCTGCAGCCTGCTTTTCATATTTTTCAAC
Mx2-RRR-11-13-AAA-SfiI-F	ctctggccgagagggccATGTCTAAGGCCCACAAGCCTTGGCCCTACGCGGCGGCAAGT
Mx1-Mx2N91-F	CGAGAACAACCTGTACAGCCAGTATGAGGAGAAGGTGCGC
Mx1-Mx2N91-R	GCGCACCTTCTCCTCATACTGGCTGTACAGGTTGTTCTCG
Mx1-nostop-SfiI-R	ctctggccagagaggccACCGGGGAACTGGGCAAGCCG
Mx1-SfiI-F	ctctggccgagagggccATGGTTGTTTCCG
Mx2-R455D-F	CCCGTTTATACAACAAAATCGACGAGGATTTTAAAAACTGGG
Mx2-R455D-R	CCCAGTTTTTAAAATCCTCGTCGATTTTGTTGTATAAACGGG
Mx2-delN25-SfiI-F	ctctggccgagagggccATGAATTCCTTCC
Mx1-myc-Sfi1-R	ctctggccagagaggccTCACAGATCCTCTTCAGAGATGAGTTTCTGCTCACCGGGGAACTGGGCAAGCCG
Mx2-SfiI-RRR-AAA-F2	CTCTGGCCGAGAGGGCCATGTCTAAGGCCCACAAGCCTTGGCCCTACGCGGCGGCAAGTCAATTTT CTTCTCG
Mx2-SfiI-SSS-AAA-F	ctctggccgagagggccATGTCTAAGGCCCACAAGCCTTGGCCCTACCGGAGGAGAGCTCAATTTGCTGCT CGAAAATACCTG
Mx2-SfiI-SSS-DDD-F	ctctggccgagagggccATGTCTAAGGCCCACAAGCCTTGGCCCTACCGGAGGAGAGATCAATTTGATGATC GAAAATACCTG
Mx2-T151D-F	GCGGAATCGTAGACAGGTGTCCGC
Mx2-T151D-R	GCGGACACCTGTCTACGATTCCGC
Mx1-K83A-F	CCAGAGCTCGGGCGCGAGCTCCGTG
Mx1-K83A-R	CACGGAGCTCGCGCCCGAGCTCTGG
Mx1-T103A-F	GCGGGATCGTGGCCAGATGCCCG
Mx1-T103A-R	CGGGCATCTGGCCACGATCCCGC
Mx1-T103D-F	GCGGGATCGTGGACAGATGCCCG
Mx1-T103D-R	CGGGCATCTGTCCACGATCCCGC
**TRIM5-fusions**	
CypA R69H F	CTTCACACACCATAATGGCACTGGT
CypA R69H R	ACCAGTGCCATTATGGTGTGTGAAG
CypA NotI F	ataagaatgcggccgccATGGTCAACCCCACCGTG
CypA SalI R	ataagaatgtcgactcaTTCGAGTTGTCCACAGTCAGC
CypA D66N F	GGTGGTAACTTCACACGCCATAATG
CypA D66N R	CATTATGGCGTGTGAAGTTACCACC
CypA (NH) F	GGTGGTAACTTCACACACCATAATG
CypA (NH) R	CATTATGGTGTGTGAAGTTACCACC
**AAV**	
AAV NheI F	TTCCTGCGGCCGCAGCATAGCTAGC
AAV XhoI R	CGCTCGGTCCGCACAATTCCTCGAG
**HIV-1 CA mutants**	
CapNM F	GTA AGA AAA AGG CAC AGC AAG CGG CCG CTG
CapNM R	CTT GGC TCA TTG CTT CAG CCA AAA CGC GTG
N57A F	AACACCATGCTAGCCACAGTGGGG
N57A R	CCCCACTGTGGCTAGCATGGTGTT
N57D F	AACACCATGCTAGACACAGTGGGG
N57D R	CCCCACTGTGTCTAGCATGGTGTT
**CypA-mutant cell verification**	
CypA Intron1 F2 (In1 F2)	TCTAAACTTGGCGCGTGTCT
CypA Intron 4 R (In4 R)	TTCAACCACCCAGCTAAGGG
CypA Intron 1 F (In1 F)	AAGAGAAGTGCACACGGATACT
CypA Intron 4 R2 (In4 R2)	GTCAGGTGGTTAGTGTGCCA
