# Supplementary figures and images for: Virus specificity and nucleoporin requirements for MX2 activity are affected by GTPase function and capsid-CypA interactions
Source: PLoS Pathog. 2024 Mar 21;20(3):e1011830. doi: 10.1371/journal.ppat.1011830 (PMC10986937; doi:10.1371/journal.ppat.1011830)

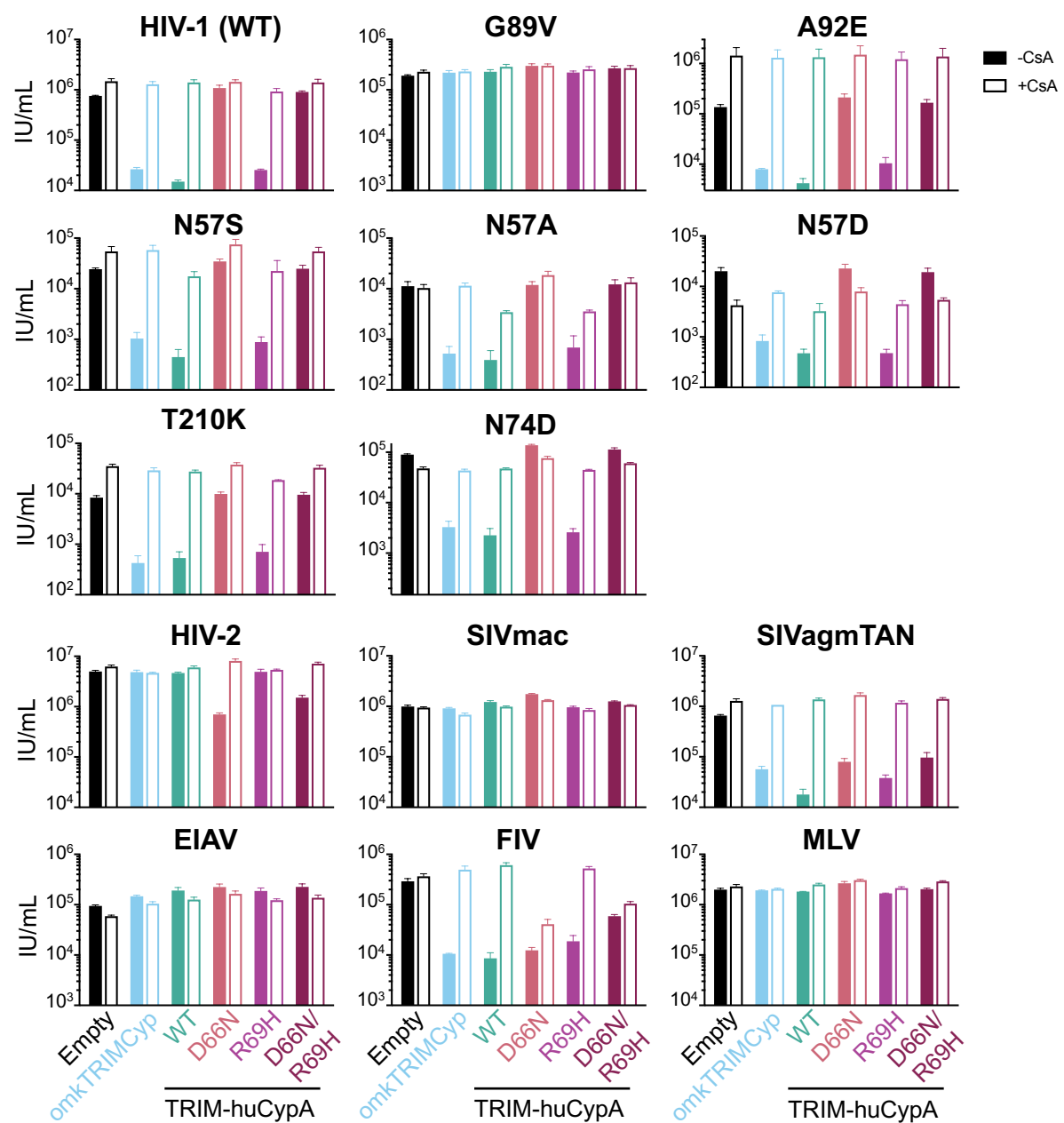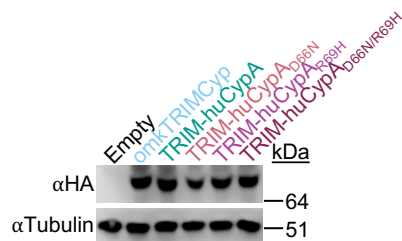

Supplement: S1 Fig — A) Infectivity of GFP reporter viruses on HeLa cells stably expressing control empty vector, owl monkey TRIMCyp (owmTRIMCyp), or chimeras of the TRIM5 N-terminal domain with human cyclophilin A (huCypA), or human CypAD66N, CypAR69H, CypAD66N/R69H mutants. Titers are represented as mean + sem of infectious units (IU) per mL, n = 3 technical replicates representative of five independent experiments. Statistical analysis in S1 File. B) Expression of the TRIM fusion proteins tagged with HA and tubulin loading control in stable cell lines used in (A). (PDF) [file ppat.1011830.s003.pdf]

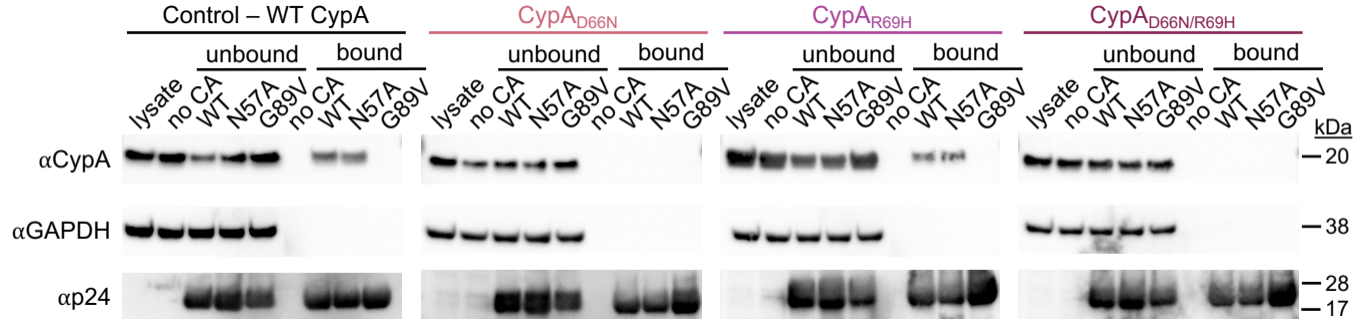

Supplement: S3 Fig — HIV-1 WT, N57A, or G89V CA tubes were assembled in vitro and incubated with lysates from control or CypA-mutant HT1080 cells as indicated. The reaction mixtures were subjected to centrifugation to separated pulled-down (bound) fractions from unbound proteins in supernatants. Lane 1: cellular lysates; Lane 2: supernatant from control experiments without CA tubes; Lanes 3–5: supernatants after incubating cellular lysates with CA tubes; Lane 6: pulled-down fraction from control experiment in the absence of CA tubes; Lanes 7–10: proteins bound to CA tubes. (PDF) [file ppat.1011830.s005.pdf]

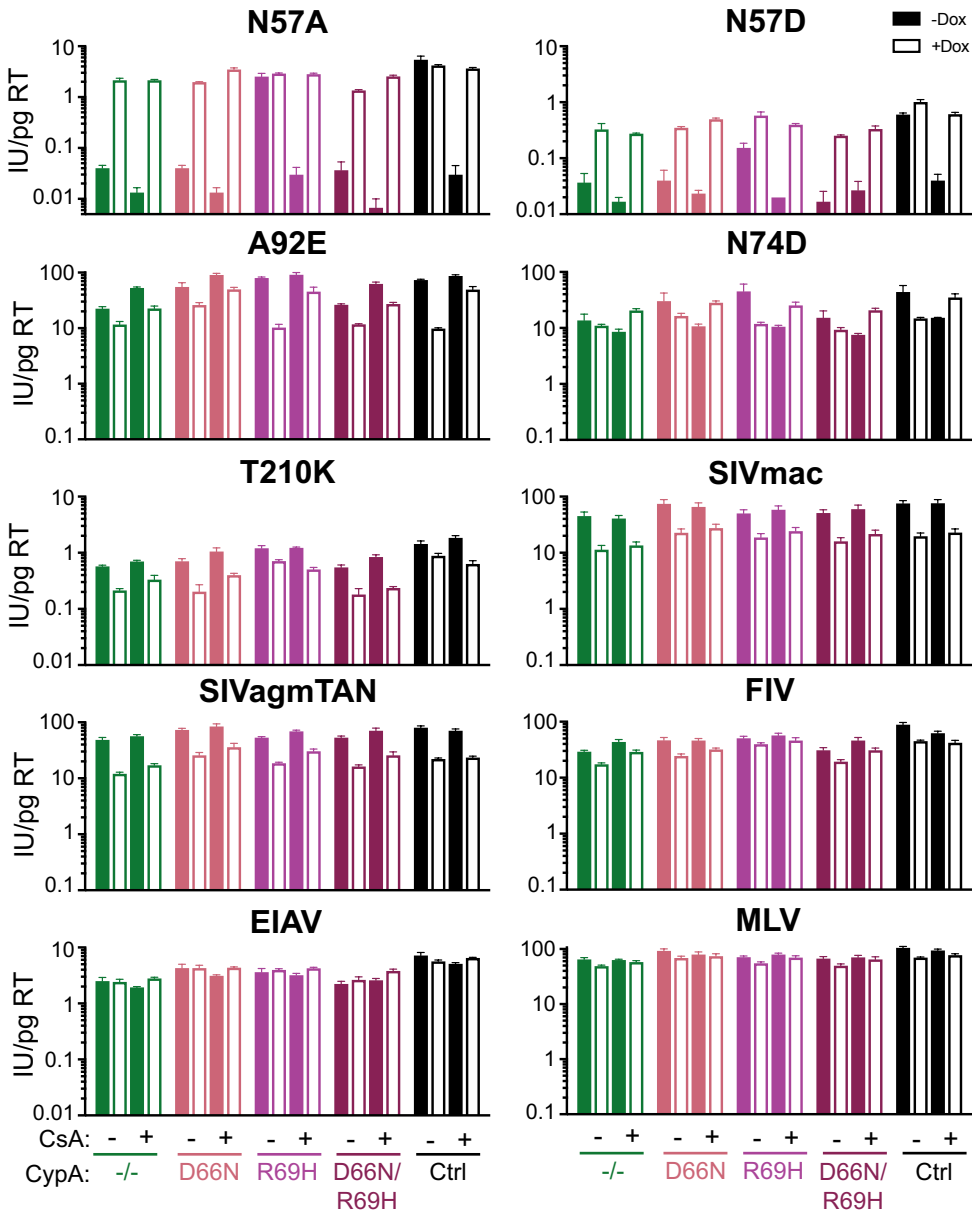

Supplement: S4 Fig — Infection of control or CypA-mutant HT1080 cells (one representative clone each) expressing doxycycline-inducible MX2 in the presence (open bars) or absence (filled bars) of doxycycline (Dox) and present or absence of CsA with indicated GFP reporter viruses. Titers are represented as mean + sem of infectious units (IU) per pg of reverse transcriptase (RT), n≥6 technical replicates combined from two-three independent experiments. Statistical analysis in S1 File. (PDF) [file ppat.1011830.s006.pdf]

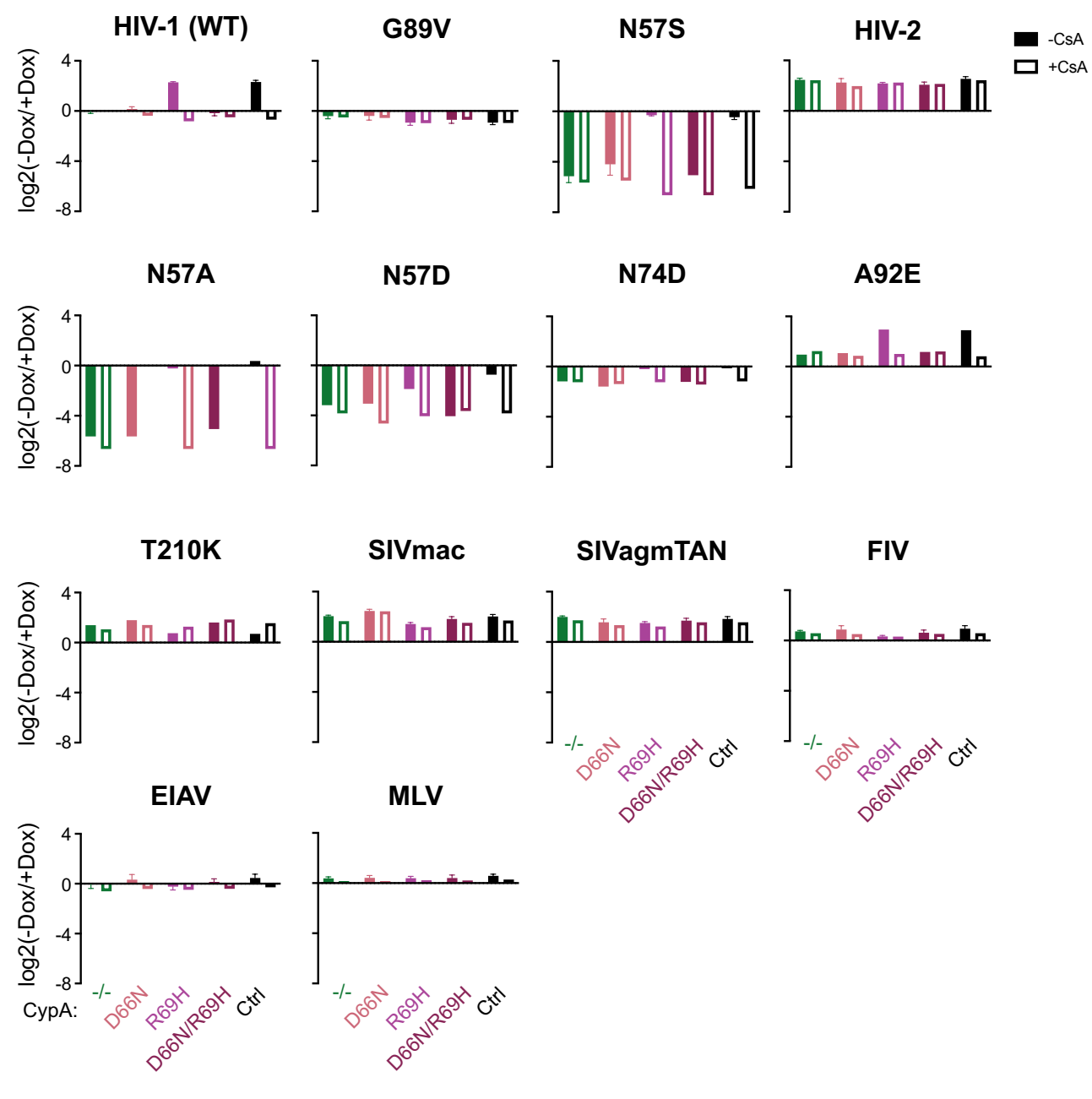

Supplement: S5 Fig — Data from Figs 1 and S4 shown as a ratio (fold change) of -Dox (-MX2)/+Dox (+MX2) in the presence (open bars) or absence (filled bars) of CsA. Average fold change calculated from three technical replicates per experiment; shown is mean + sem of log2(fold change) from two-three independent experiments. (PDF) [file ppat.1011830.s007.pdf]

**A**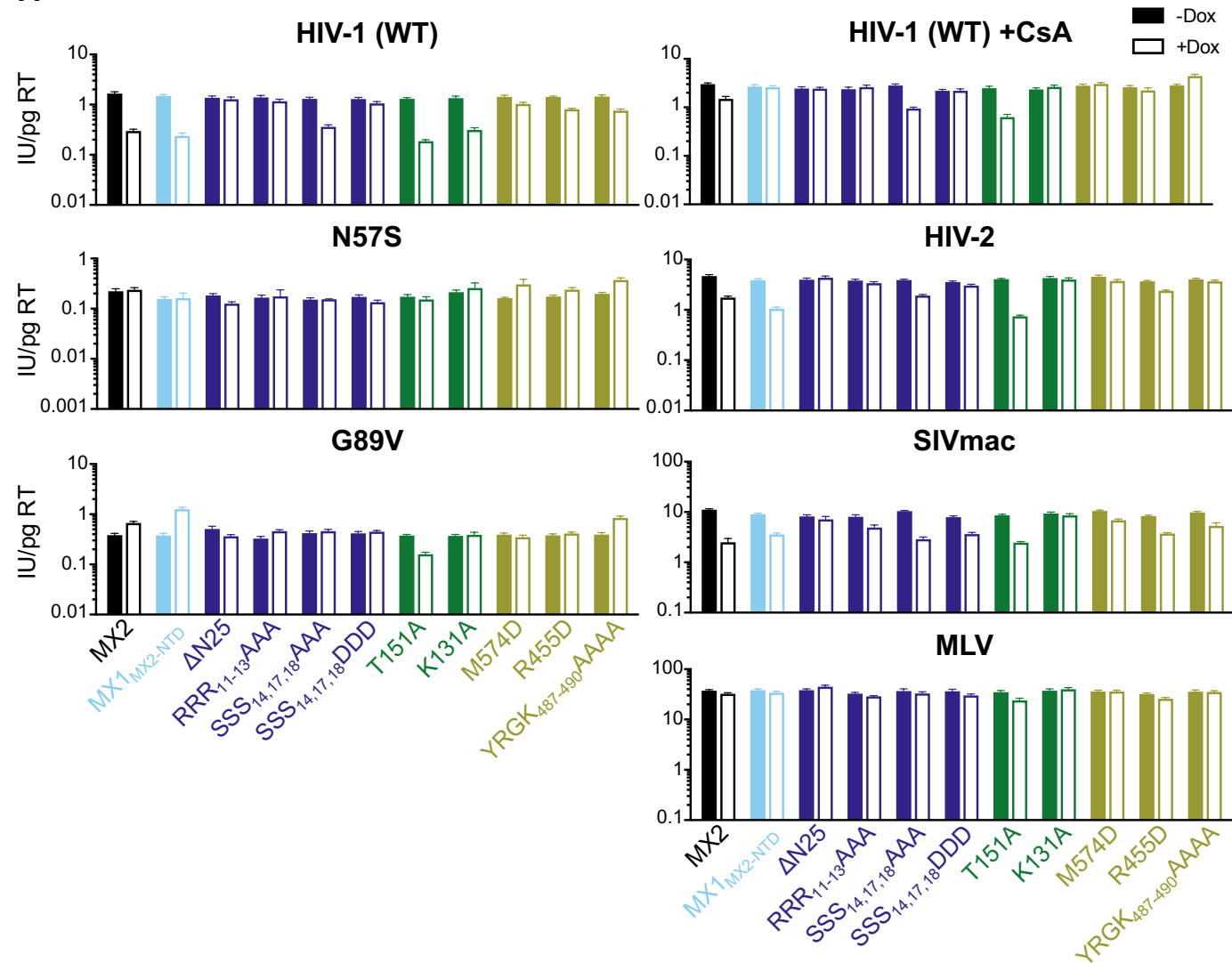**B**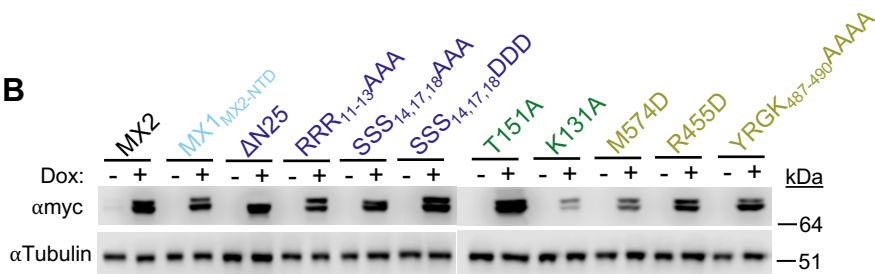

Supplement: S6 Fig — A) Infectivity of GFP reporter viruses in HeLa cells expressing doxycycline-inducible C-terminally myc-tagged MX2, MX2 mutants, or MX1MX2-NTD in the presence (open bars) and absence (filled bars) of doxycycline (Dox). MX2 mutants are color-coded by domain/effect as in Fig 2A. Cells were infected in the presence of CsA where indicated. Titers are represented as mean + sem of infectious units (IU) per pg of reverse transcriptase (RT), n≥8 technical replicates combined from two-six independent experiments. Statistical analysis in S1 File. B) Western blot analysis of doxycycline-inducible MX2-myc and tubulin loading control. (PDF) [file ppat.1011830.s008.pdf]

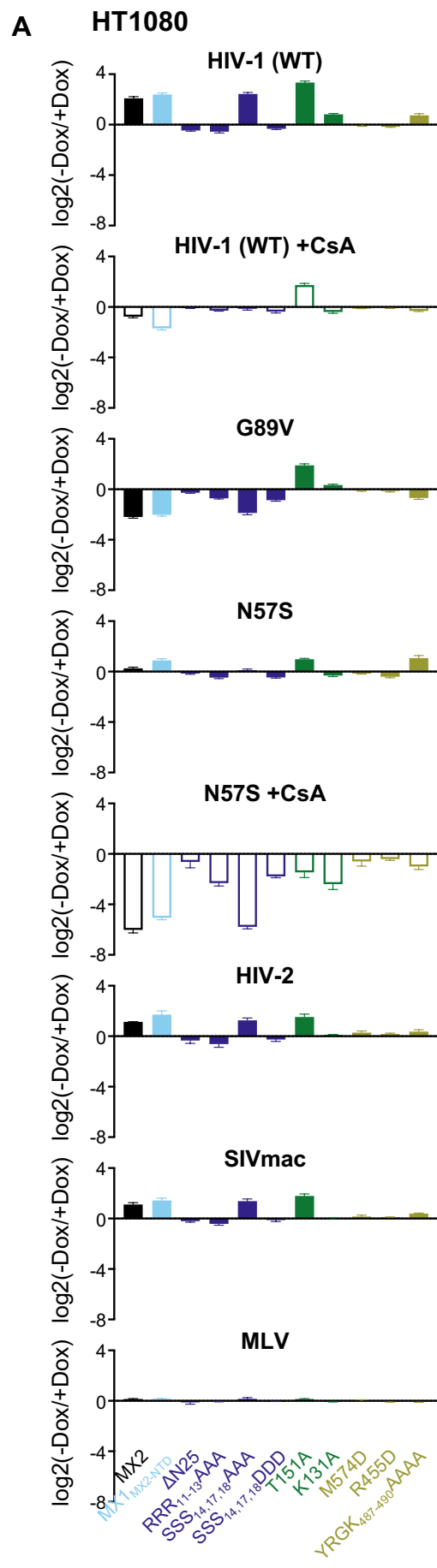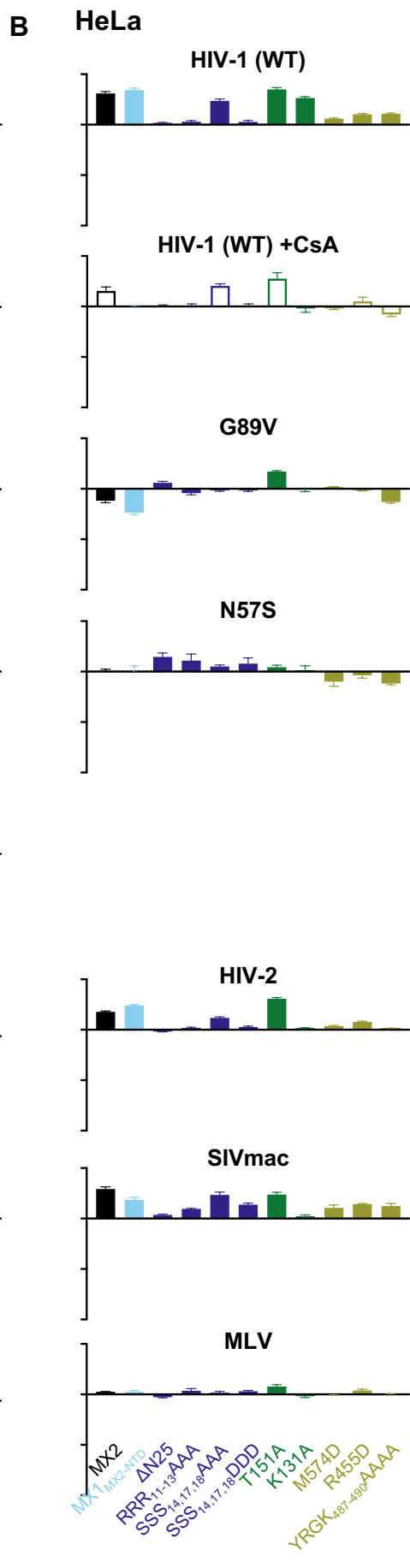

Supplement: S7 Fig — Data from Figs 3 and S6 shown as a ratio (fold change) of -Dox (-MX2)/+Dox (+MX2). Average fold change calculated from four technical replicates per experiment; shown is mean + sem of log2(fold change) from two-six independent experiments. (PDF) [file ppat.1011830.s009.pdf]

**A**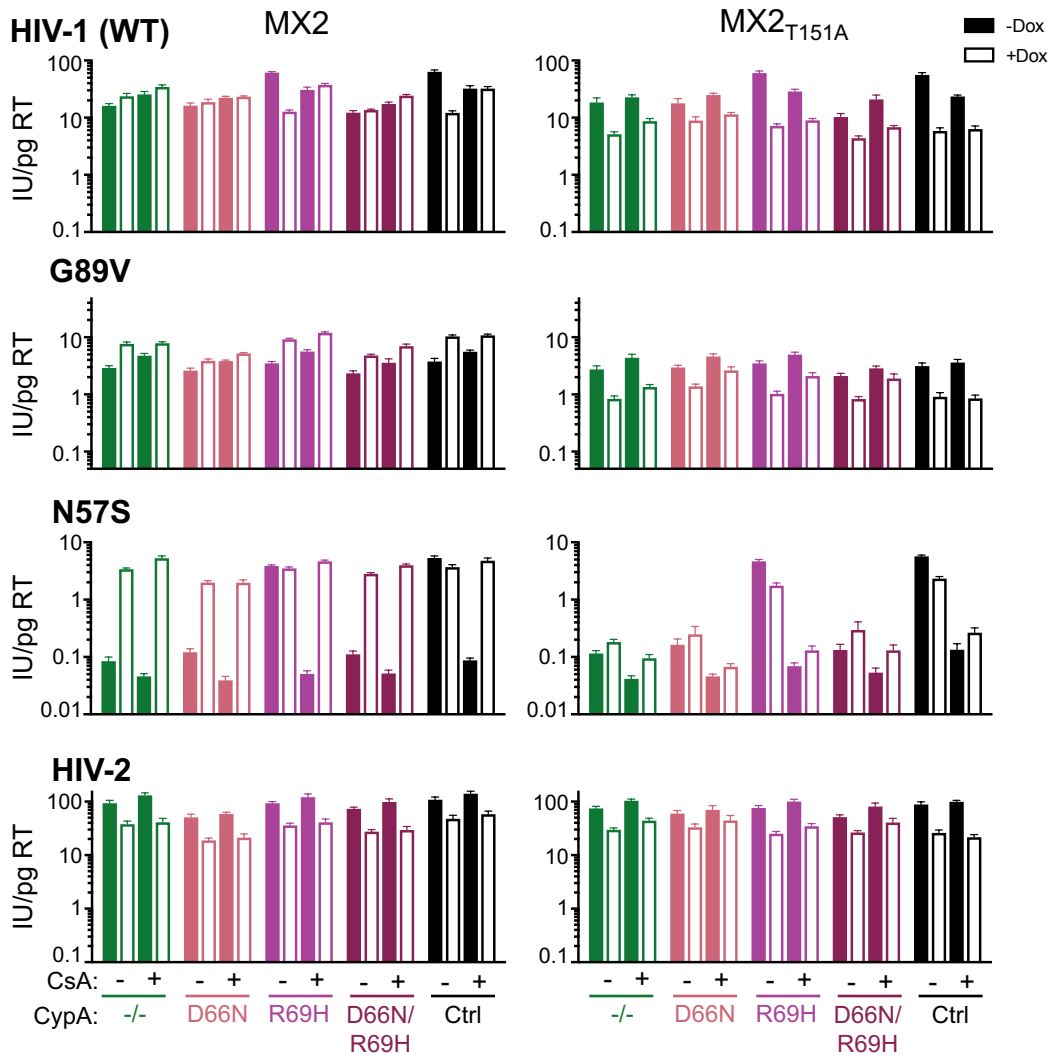**B**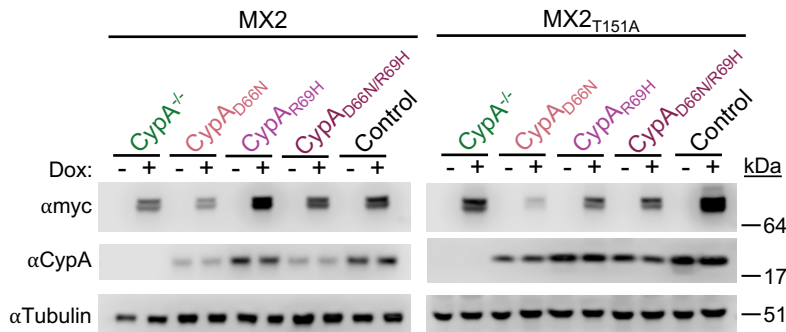

Supplement: S8 Fig — A) Infection of control and CypA-mutant HT1080 cell clones (one representative clone each) stably transduced with doxycycline-inducible myc-tagged MX2 (left) or MX2T151A (right) in the presence (open bars) or absence (filled bars) of doxycycline and presence or absence of CsA with the indicated GFP reporter viruses. Titers are represented as mean + sem of infectious units (IU) per pg of reverse transcriptase (RT), n≥12 technical replicates combined from five independent experiments. Statistical analysis in S1 File. B) Western blot analysis of doxycycline-inducible MX2-myc, CypA, and tubulin loading control in the indicated cell clones. (PDF) [file ppat.1011830.s010.pdf]

MX2

MX2<sub>T151A</sub>

■ -CsA

□ +CsA

HIV-1 (WT)

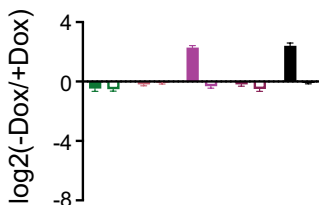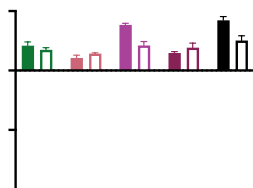

G89V

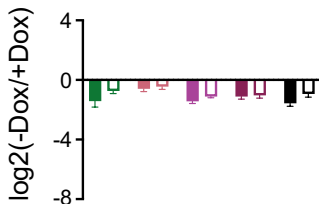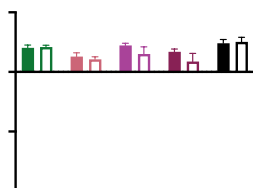

N57S

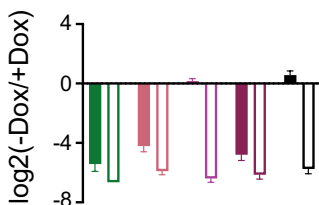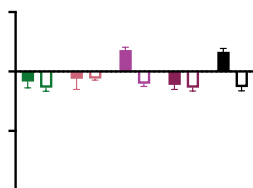

HIV-2

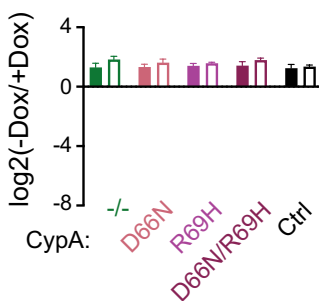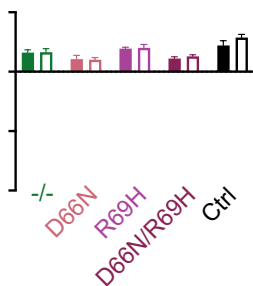

Supplement: S9 Fig — Data from S8 Fig shown as a ratio (fold change) of -Dox (-MX2)/+Dox (+MX2) in the presence (open bars) or absence (filled bars) of CsA. Average fold change calculated from three technical replicates per experiment; shown is mean + sem of log2(fold change) from five independent experiments. (PDF) [file ppat.1011830.s011.pdf]

**A**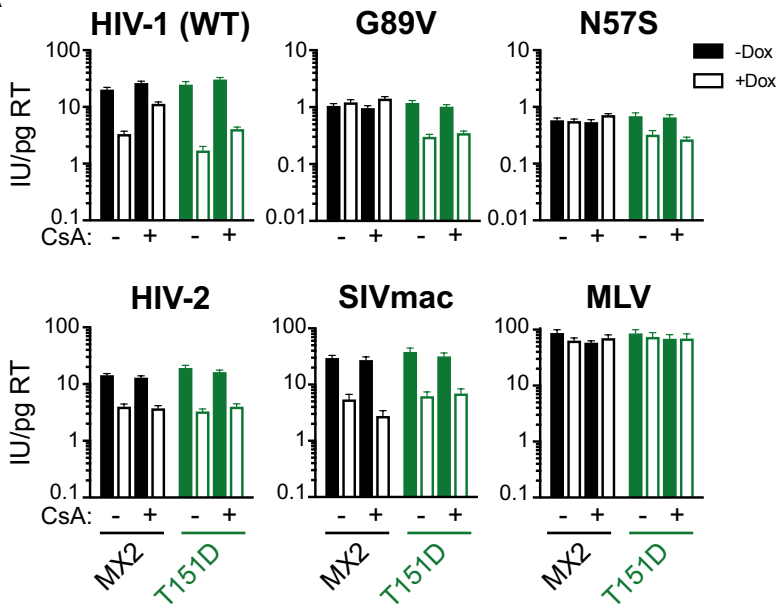**B**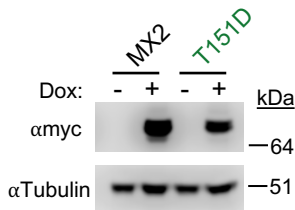

Supplement: S10 Fig — A) Infection HeLa cells stably transduced with doxycycline-inducible myc-tagged MX2 or MX2T151D in the presence (open bars) or absence (filled bars) of doxycycline and presence or absence of CsA with the indicated GFP reporter viruses. Titers are represented as mean + sem of infectious units (IU) per pg of reverse transcriptase (RT), n≥6 technical replicates combined from two-seven independent experiments. Statistical analysis in S1 File. B) Western blot analysis of doxycycline-inducible MX2-myc and tubulin loading control. (PDF) [file ppat.1011830.s012.pdf]

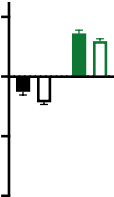

Supplement: S11 Fig — Data from S8 Fig. shown as a ratio (fold change) of -Dox (-MX2)/+Dox (+MX2) in the presence (open bars) or absence (filled bars) of CsA. Average fold change calculated from three technical replicates per experiment; shown is mean + sem of log2(fold change) from five independent experiments. (PDF) [file ppat.1011830.s013.pdf]

**A**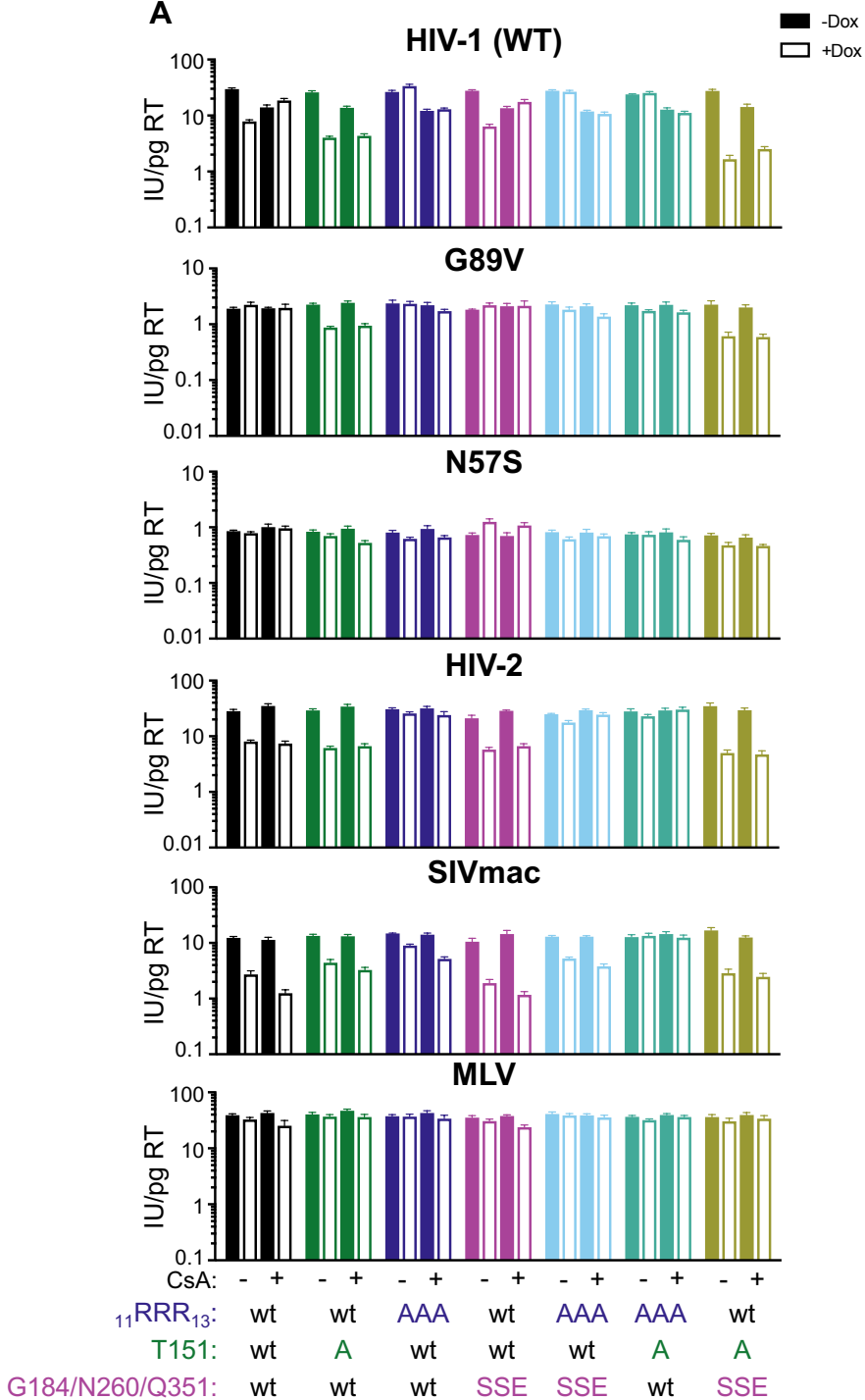**B**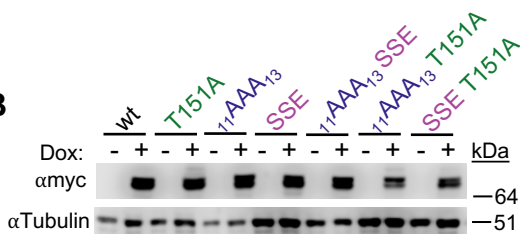

Supplement: S12 Fig — A) Infection of HeLa cells stably transduced with doxycycline-inducible myc-tagged MX2 with or without mutations in the N-terminal triple-arginine motif (11AAA13), T151A, or CA-binding residues in the GTPase domain (SSE) in the presence (open bars) or absence (filled bars) of doxycycline and presence or absence of CsA with the indicated GFP reporter viruses. Titers are represented as mean + sem of infectious units (IU) per pg of reverse transcriptase (RT), n = 9 technical replicates combined from three independent experiments. Statistical analysis in S1 File. B) Western blot analysis of doxycycline-inducible MX2-myc and tubulin loading control. (PDF) [file ppat.1011830.s014.pdf]

A

HT1080

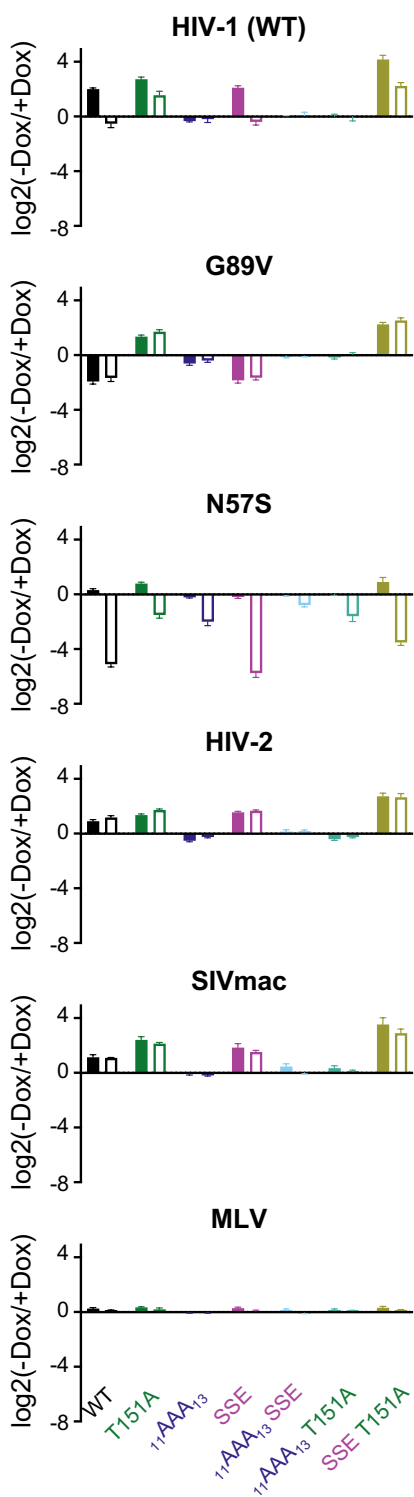

B

HeLa

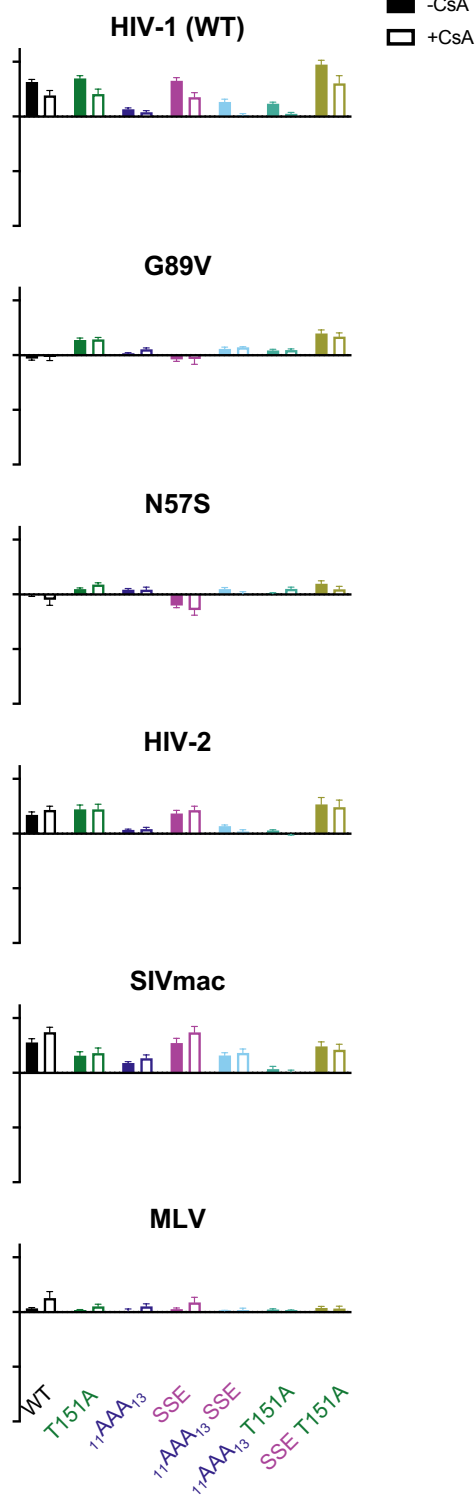

Supplement: S13 Fig — Data from Figs 5 and S12 shown as a ratio (fold change) of -Dox (-MX2)/+Dox (+MX2) in the presence (open bars) or absence (filled bars) of CsA. Average fold change calculated from three technical replicates per experiment; shown is mean + sem of log2(fold change) from three-five independent experiments. 11AAA13 = RRR11-13AAA; SSE = G184S/N260S/Q351E. (PDF) [file ppat.1011830.s015.pdf]

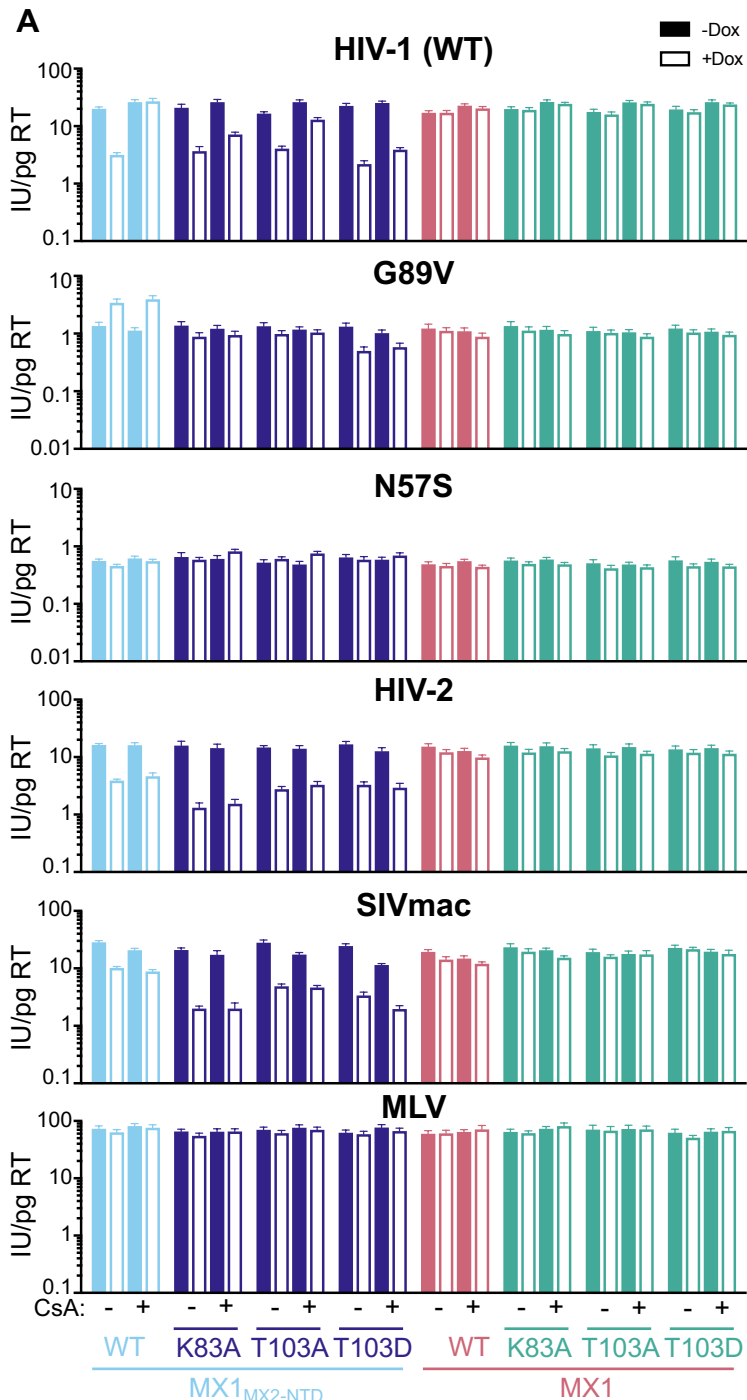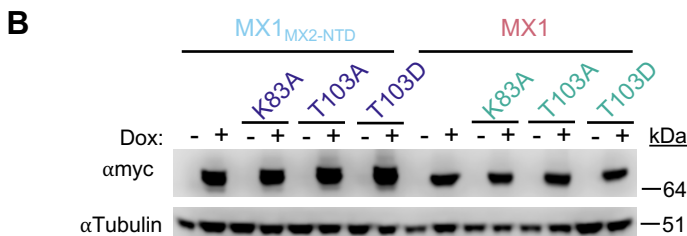

Supplement: S14 Fig — A) Infection of HeLa cells stably transduced with doxycycline-inducible myc-tagged MX1 or MX1MX2-NTD with the indicated mutations in the presence (open bars) or absence (filled bars) of doxycycline and presence or absence of CsA with the indicated GFP reporter viruses. Titers are represented as mean + sem of infectious units (IU) per pg of reverse transcriptase (RT), n≥9 technical replicates combined from three-seven independent experiments. Statistical analysis in S1 File. B) Western blot analysis of doxycycline-inducible MX1MX2-NTD or MX1-myc and tubulin loading control. (PDF) [file ppat.1011830.s016.pdf]

**A****B****HeLa**

■ -CsA

□ +CsA

**HT1080**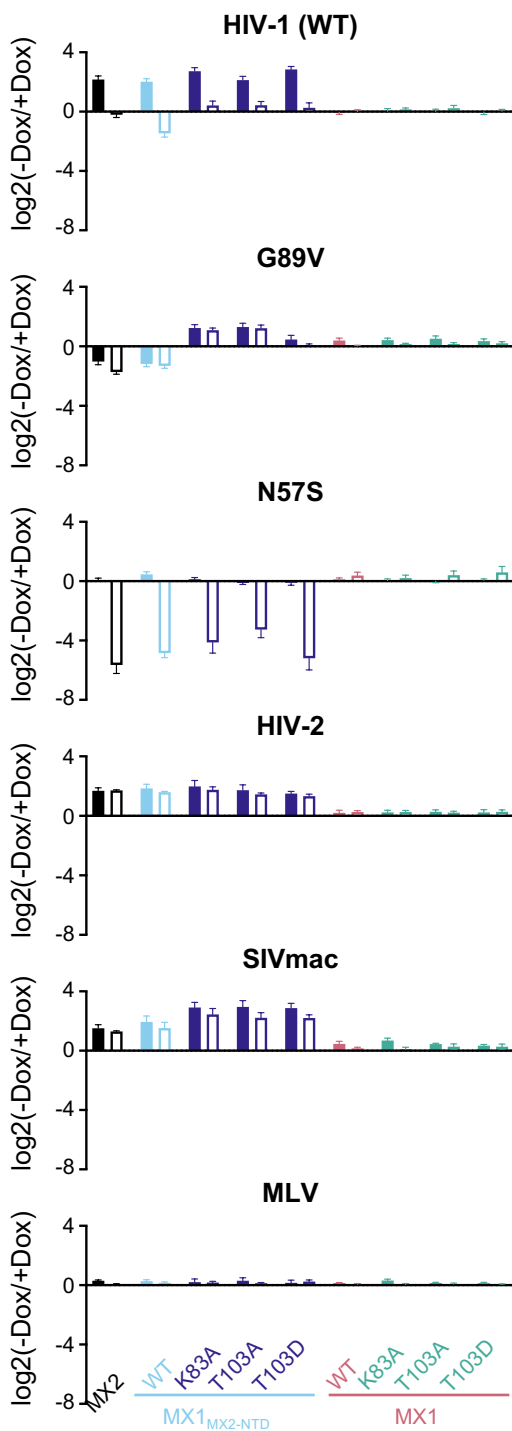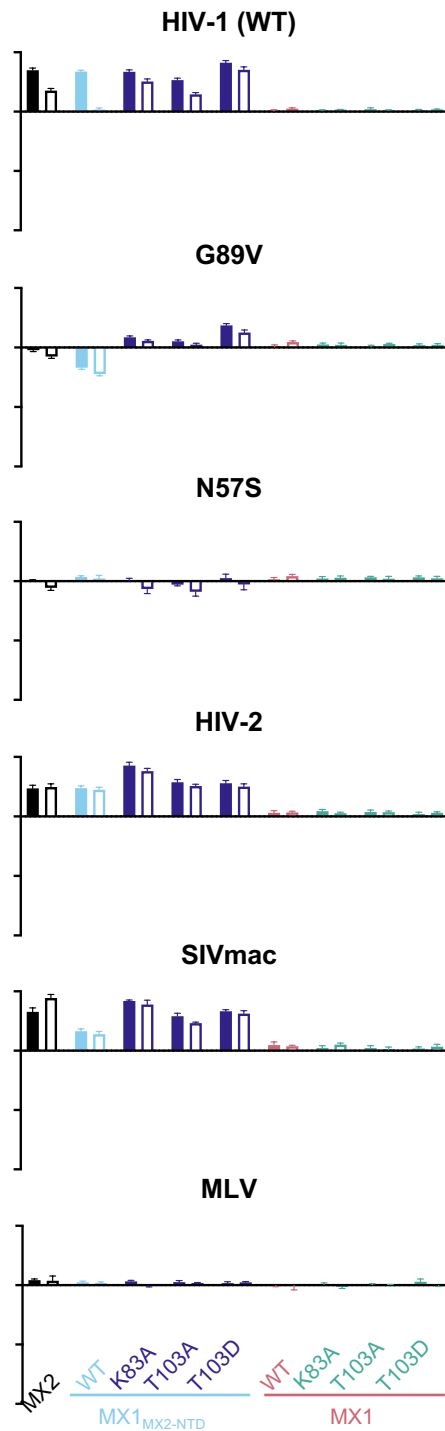

Supplement: S15 Fig — Data from Figs 6 and S14 shown as a ratio (fold change) of -Dox (-MX2)/+Dox (+MX2) in the presence (open bars) or absence (filled bars) of CsA. Average fold change calculated from three technical replicates per experiment; shown is mean + sem of log2(fold change) from three-seven independent experiments. (PDF) [file ppat.1011830.s017.pdf]

**A**

Transfect HT0180 stably transduced  
with Dox-inducible **MX2** or **MX2<sub>T151A</sub>**  
with Nup/importin siRNA

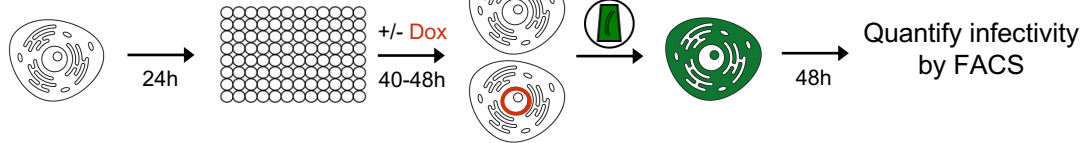**B**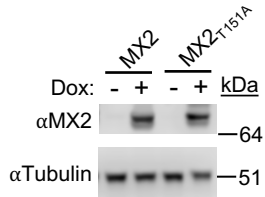

Supplement: S16 Fig — A) Experimental strategy to investigate the roles of Nups and NTRs in antiviral activity of MX2 and MX2T151A. For a detailed description, refer to the Materials and Methods. B) Western blot analysis of doxycycline-inducible MX2 and MX2T151A and tubulin loading control. (PDF) [file ppat.1011830.s018.pdf]
