# Supplementary material for: Virus specificity and nucleoporin requirements for MX2 activity are affected by GTPase function and capsid-CypA interactions
Source: PLoS Pathog. 2024 Mar 21;20(3):e1011830. doi: 10.1371/journal.ppat.1011830 (PMC10986937; doi:10.1371/journal.ppat.1011830)
Supplement: S2 Fig — A) Schematic of the PPIA (CypA) locus on Chromosome 7 with crRNA guide targeting sites intron 1 and exon 4, and PCR primers for clone screening and verification indicated. Diagram of AAV donor for homology-directed repair shown below, as well as amino acid residues 66–69 in wild-type and mutant CypA cells. B) Sequences of the wild-type locus at targeted sites, with crRNA and PAM sites indicated. Dots indicate sequences flanking those shown in detail. Allele sequences of CypA-/- clones with mismatches to wild-type sequence highlighted and deleted sequence indicated by dashes, consequence of mutations in each knock-out allele shown below. C) Sequences of wild-type and point-mutant alleles with nucleotide changes in codons and HpaI site in intron 3 indicated. D) Top: Infectivity of HIV-1 GFP reporter virus infection in control, CypA-/- (left), and CypA-mutant (right) cell clones expressing doxycycline-inducible MX2 in the presence (open bars) or absence (filled bars) of doxycycline (Dox) and presence or absence of CsA. Alleles of CypA in knock-out clones detailed in (B) are indicated. Titers are represented as mean + sem of infectious units (IU) per pg of reverse transcriptase (RT); left: n≥6 technical replicates combined from three independent experiments; right: n≥3 technical replicates representative of four independent experiments. Statistical analysis in S1 File. Bottom: western blot analysis of doxycycline-inducible MX2, CypA, and tubulin loading control in the indicated cell clones. (PDF) [file ppat.1011830.s004.pdf]

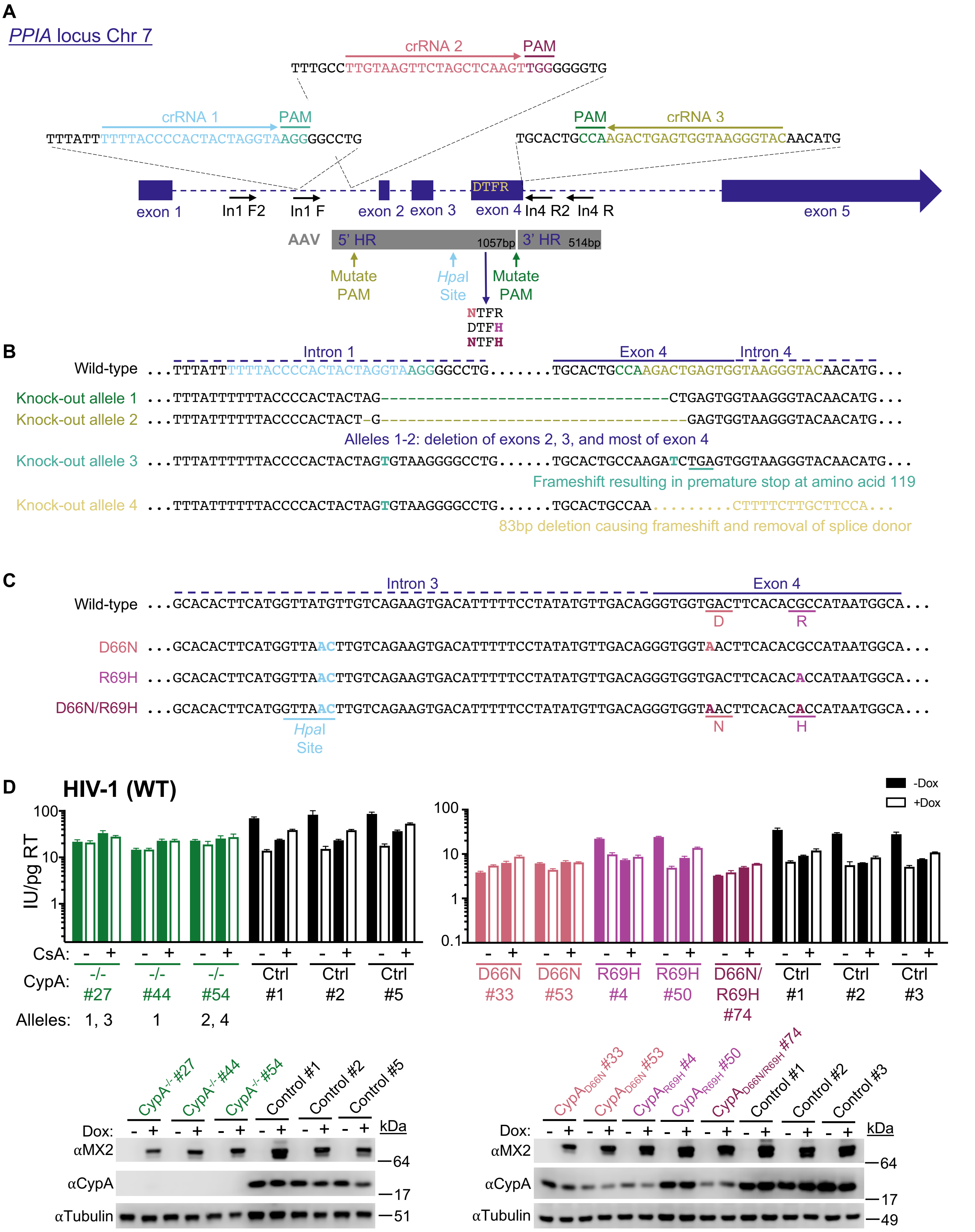
A
PPIA locus Chr 7
crRNA 2
PAM
TTTGCCTTGTAAGTTCTAGCTCAAGTTGGGGGGTG
crRNA 1
PAM
TTTATTTTTTACCCCACTACTAGGTAAGGGGCCTG
PAM
crRNA 3
TGCACTGCCAAGACTGAGTGGTAAGGGTACAACATG
DTFR
exon 1
In1 F2
In1 F
exon 2
exon 3
exon 4
In4 R2
In4 R
exon 5
AAV
5' HR
1057bp
3' HR
514bp
Mutate PAM
HpaI Site
Mutate PAM
NTFR
DTFH
NTFH
B
Intron 1
Exon 4
Intron 4
Wild-type ...TTTATTTTTTACCCCACTACTAGGTAAGGGGCCTG......TGCACTGCCAAGACTGAGTGGTAAGGGTACAACATG...
Knock-out allele 1 ...TTTATTTTTTACCCCACTACTAG---------------------------------CTGAGTGGTAAGGGTACAACATG...
Knock-out allele 2 ...TTTATTTTTTACCCCACTACT-G-----------------------------------GAGTGGTAAGGGTACAACATG...
Alleles 1-2: deletion of exons 2, 3, and most of exon 4
Knock-out allele 3 ...TTTATTTTTTACCCCACTACTAGTGTAAGGGGCCTG.....TGCACTGCCAAGATCTGAGTGGTAAGGGTACAACATG...
Frameshift resulting in premature stop at amino acid 119
Knock-out allele 4 ...TTTATTTTTTACCCCACTACTAGTGTAAGGGGCCTG.....TGCACTGCCAA.........CTTTTCTTGCTTCCA...
83bp deletion causing frameshift and removal of splice donor
C
Intron 3
Exon 4
Wild-type ...GCACACTTCATGGTTATGTTGTCAGAAGTGACATTTTTCCTATATGTTGACAGGGTGGTGACTTCACACGCCATAATGGCA...
D
R
D66N ...GCACACTTCATGGTTAACTTGTCAGAAGTGACATTTTTCCTATATGTTGACAGGGTGGTAACTTCACACGCCATAATGGCA...
R69H ...GCACACTTCATGGTTAACTTGTCAGAAGTGACATTTTTCCTATATGTTGACAGGGTGGTGACTTCACACACCATAATGGCA...
D66N/R69H ...GCACACTTCATGGTTAACTTGTCAGAAGTGACATTTTTCCTATATGTTGACAGGGTGGTAACTTCACACACCATAATGGCA...
HpaI Site
N
H
D
HIV-1 (WT)
IU/pg RT
100
10
1
0.1
-Dox
+Dox
CsA:
CypA:
-/- #27
-/- #44
-/- #54
Ctrl #1
Ctrl #2
Ctrl #5
Alleles:
1, 3
1
2, 4
D66N #33
D66N #53
R69H #4
R69H #50
D66N/R69H #74
Ctrl #1
Ctrl #2
Ctrl #3
CypA-/- #27
CypA-/- #44
CypA-/- #54
Control #1
Control #2
Control #5
Dox:
kDa
αMX2
αCypA
αTubulin
64
17
51
CypA D66N #33
CypA D66N #53
CypA R69H #4
CypA R69H #50
CypA D66N/R69H #74
Control #1
Control #2
Control #3
49
