# Supplementary material for: Virus specificity and nucleoporin requirements for MX2 activity are affected by GTPase function and capsid-CypA interactions
Source: PLoS Pathog. 2024 Mar 21;20(3):e1011830. doi: 10.1371/journal.ppat.1011830 (PMC10986937; doi:10.1371/journal.ppat.1011830)
Supplement: Attachment — Submitted filename: Layish B Response to Reviewers.pdf [file ppat.1011830.s019.pdf]

Part I - Summary

Please use this section to discuss strengths/weaknesses of study, novelty/significance, general execution and scholarship.

Responses to reviewer comments are in blue italic typeface.

We thank the reviewers for their time and thoughtful critique of our manuscript. We have made every effort to address the concerns, clarify points of confusion, and/or correct errors in the initial submission and believe that we have addressed their concerns and improved the manuscript. Detailed responses to the individual comments are in blue text below.

Reviewer #1: In this article Layish and colleagues investigate some of the determinants governing the anti-lentiviral activity of human MX2. They have performed a large array of experiments analysing the effect of MX2 mutants on many lentiviruses and in different conditions. They have provided important pieces of evidence, nonetheless the demonstration that the effect of CSA treatment on HIV-1 infected cells is directly due to its effect on the CA-CypA interaction. They follow on showing how the regulation of MX2's antiviral activity involves the association of CypA with the viral capsid, the GTPase activity of the protein, the presence of specific nuclear pore components on a cell line-based dependent manner. This article provides valuable information on the antiviral activity of MX2 and aids to understand the highly complex nature of its regulation by viral and cellular factors. However, I think the article is missing an overall conclusion (a punch line) on its findings. While the data clearly shows the important role of CypA and GTP binding on the regulation of MX2's antiviral activity, with the interesting comparison of wild type and N57S viruses, it lacks a hypothesis or model that tries to tie together the findings. How do the authors think everything comes at play to regulate MX2's activity? Is GTP binding changing how or even where MX2 interacts with CA and that makes it "CypA independent", and down the line lead to the engagement of different nuclear pore proteins? Does MX2 has a GTPase-dependent and a GTPase-independent inhibitory mechanism?

We appreciate the concern over the lack of an over-arching conclusion about how GTPase activity regulated MX2 function. We have added discussion of a model that ties these findings together with the existing literature (Lines 426-438). Unfortunately, as the reviewer notes, myriad factors affect MX2 function in complex ways, making it impossible to draw definitive conclusions regarding how all the players come together. We hope that this report advances our understanding of these complexities and that the additions to the discussion tie this particular investigation together.

Reviewer #2: The manuscript by Layish et al. describes a comprehensive study to determine the specificity of Mx2 restriction in HT1080 and HeLa cells to different viral isolates and by modification of endogenous CypA by Knock-In by Cas9 and/or CsA presence.

The manuscript answers many unknowns in the topic and is very comprehensive and complete in the approach. In general the results support the conclusions of the discussion. The description of GTPase deficient Mx2 mutants together with measurement of CA determinants, CypA/CsA activity further sheds light on how Mx2 targets incoming HIV-1 and articulates with other cell proteins.

Some of the discussion should accommodate other possibilities on indirect effects on Mx2 restriction by CypA capability to isomerize other host proteins as the only direct binding data shown in the article is the nanotube data in Fig S3 (It is correctly stated in results lines 150 to 153, but not in the discussion). The relationship with TRIM5alpha should also be discussed.

We have added text to the discussion regarding both CypA (Lines 430-435) and TRIM5α (Lines 417-425)

There are also several items that should be improved or corrected as the data is extensive making it certainly hard to follow to a broader audience.

We have made every effort to provide the data in a more digestible format as suggested by this reviewer below by the inclusion of figures specifically showing restriction/enhancement by MX2.

Part II – Major Issues: Key Experiments Required for Acceptance
Please use this section to detail the key new experiments or modifications of existing experiments that should be absolutely required to validate study conclusions.

Generally, there should be no more than 3 such required experiments or major modifications for a "Major Revision" recommendation. If more than 3 experiments are necessary to validate the study conclusions, then you are encouraged to recommend "Reject".

Reviewer #1: (No Response)

Reviewer #2: Line 115: HIV-1WT infection was potently inhibited in TRIM-huCypA(R69H) and not D66N and D66N/R69H. I believe this to be an editing error as the conclusions are in accordance to the data in Fig 1 and Fig S1.

This was an error and has been corrected

Line 122 (and others): specify and clarify on when and how many clones were used in the experiments, it is not clear if the data in the figures are a mix of the several described clones in FigS2, only the best one or if there is divergence in their phenotypes.

We have clarified in the Methods section (Lines 529-531) that representative clones were selected. Importantly, there is minimal clonal variability in the HT1080 cells, and no significant differences were observed in the clones. The figures utilizing the clones all show data from the same individual clones, although experiments were conducted with all clones.

Fig 7: The results of Nup85, Sec13 and other depletions to Mx2 restriction are not described. The authors should discuss and hypothesize why MX2 does not resctrict upon depletion but but MX2 T151A does (for HIV-1 WT).

We note on Lines 326-327 and Lines 338-341 that depletion of components of the Nup107 subcomplex (including NUP85 and SEC13) have differential effects on MX2 and $MX2_{T151A}$ activity. The discussion addresses the possible explanations for these differences (eg. altered cellular localization of $MX2_{T151A}$, altered conformation of $MX2_{T151A}$ that affects interactions with Nups, and/or altered interactions of $MX2_{T151A}$ with the HIV-1 CA that affect downstream interactions with Nups. Due to the pleiotropic effects of Nup depletion, additional conclusions regarding the role of individual Nups cannot be drawn from these experiments.

-All data is normalized to IU/pgRT, as changing HIV CA results in very dramatic changes in infectivity, it is possible that some results are more susceptible to saturation than others. Authors should discuss this possibility and address it.

All infections were conducted to ensure values were within the linear range of infection, so none of the experiments shown are susceptible to saturation. The normalization to IU/pg of RT allows us to combine data using virus-preps produced on different days so that biological replicates can be presented. We have added clarification on this to the Methods section (Lines 551-553).

Part III – Minor Issues: Editorial and Data Presentation Modifications
Please use this section for editorial suggestions as well as relatively minor modifications of existing data that would enhance clarity.

Reviewer #1: 1. Lines 114-116 say “As expected, HIV-1WT infection was potently inhibited (~200-fold) by TRIM-huCypA D66N and TRIM-huCypA D66N/R69H in a manner reversible by CsA treatment, while TRIM-huCypA R69H was inactive against HIV-1WT”. I think the authors mean quite the opposite, since mutant R69H is the only one inhibiting HIV-1 infection, while those having the D66N mutation do not.

Correct, this was a mistake and has been fixed (lines 118-119)

2. Lines 375-377: describes the construction of the different omkTRIM-CypA plasmids as having “the N-terminal domains (RIGN, B-Box and coiled coil) from owl monkey (omk) TRIMCyp followed by an HA tag and cloning sites (NotI/SalI) that allowed introduction of various proteins”. As it is stated one would think that the constructs go as TRIM-HA-CypA, while they are HA-TRIM-CypA. Therefore, I believe it is best to say that the HA tag is followed by the N-terminal domains from omkTRIMCyp.

The reviewer has read this correctly, the constructs are TRIM-HA-CypA (not HA-TRIM-Cyp). This is the same system used in PMID: 24130490 and 31048506.

3. Lines 133-135: I find the wording a bit confusing. My first impression was that CSA treatment reduced levels of infectivity and a lack of MX2 sensitivity in CypA-/-, CypAD66N, and CypAD66N/R69H. I now know that what they point is that, in the absence of CSA, those CypA proteins reduced levels of infectivity and a lack of MX2 sensitivity, as addition of CSA does for wt CypA. I think rewriting this sentence to make it clearer would improve the manuscript.

We have changed the wording to improve the clarity (Line 142)

4. Line 177: an “in” is missing between CSA and HT1080. Also, in Fig S5 (HeLa cells) the antiviral activity of MX1(NTD_MX2) is very much cancelled by CSA.

Correct, we have added an “in”, and rephrased our description of the phenotype of $MX1_{MX2\text{-}NTD}$ (Lines 184-191)

5. Line 185: I think the sentence GTP-binding deficient mutant K131A makes clearer this mutant does not bind GTP.

Agreed, we have changed the wording here (Line 205)

6. Lines 186-187: I fail to understand the K131A data. It seems to be a slight reduction on infection in Fig 3. In fact, if I am understanding the S1 statistical data correctly, this reduction is of 2.605, and significant. Therefore, K131A does inhibit HIV-1 infection in HT1080 cells, and therefore the sentence in the following lines about GTP binding being cell-type dependent wouldn’t make sense. This issue arises again in lines 336-337.

We have modified the text to clarify that the K131A mutant is much less potent in HT1080 cells as compared to HeLa cells (Lines 206-207 and 389)

7. In Fig 3A, the effect of CSA with MX1 (NTD_MX2) is quite remarkable, the increase in HIV-1 infectivity seems to be ~3x, higher than any other protein tested. Do the authors think this is due to some MX1 determinant absent in MX2?

We agree this is an interesting phenotype and that there must be determinants within MX1 and we have added to/modified the text to discuss this (Lines 184-186 and 360-361)

8. I am intrigue by the immunoblot data in Fig S6. In CypA D66N cells the level of MX2 T151A is drastically reduced compared with the other conditions, and levels of MX2 are also diminished, with tubulin levels unaffected. One could think that somehow CypA D66N is producing a drop on the amount of cellular MX2, but this trend is not seemed in Fig 1, could the authors explain this?

We suspect this is related to slightly less efficient transduction or Dox-induction rather than the CypA mutant dropping MX2 levels, since as the reviewer noted, this is not observed in the cells shown in Fig 1. Since the same effect is also not observed for the D66N/R69H double-mutant, and this is a supplemental figure, we do not think this effect has a phenotypic relevance. If the reviewer feels strongly that this is an issue, we would be inclined to remove this figure since it supports the conclusions but is not essential to the overall story.

9. What is the difference between the antiviral activity of T151A and T151D? It seems that both mutants have the same inhibitory profile.

Indeed, these two mutants have the same inhibitory profile, and we have added a statement to this point (Line 254). The purpose of including T151D was to demonstrate that GTP hydrolysis-deficiency, not phosphorylation status determines the CypA-independence of the T151A mutant, therefore a similar phenotype is expected.

10. In Fig 5, in the first lane under the bar chart where the presence or not of mutations in different MX2 positions are depicted, an indication of the amino-terminal domain is missing.

Corrected

11. I find Fig 5A too difficult to follow. The combination of 8 different conditions on a single panel, while excellent for saving space, make it too complicated to comprehend. I would suggest the authors to split them in 2 or 3 panels, maybe based on the type of mutation (i.e., NTD, T151 and G domain).

We considered breaking this figure up, but since the purpose is the combination of NTD, T151, and G domain mutants, it was impossible to split up the mutants. We hope the inclusion of the new fold-change figure (S13 Fig) makes the data more digestible.

12. Fig 6 is rather difficult to follow, especially because the most significant comparison of MX1(NTD_MX2) mutants is with the corresponding MX2 mutants, rather than MX1. Therefore, having MX1(NTD_MX2) mutants side to side with MX2 in Fig 6 would be more informative.

We have added the MX2 control to the new fold-change figure (S15 Fig). However, we feel the MX1 mutants are an important control here and that the addition of more MX2 mutants would complicate, rather than simplify the figure.

13. Also in Fig 6, how are MX1 T103A/D mutants inhibiting N57S in the presence of CSA?

These are infectivity values approaching the limit of detection and are not statistically significant (S1 File). We also hope the log2(fold change) data for these experiments shown in S15 Fig clarifies that this is not a true inhibition.

14. Fig 7: indentation of B-D) needs correction.

Corrected

15. It would be interesting to see the effect of T151A on HIV-1 T210K mutant viruses, since this residue is involved in the binding of MX2's NTD and could give some more information about the viral determinants at play on the particular capabilities of T151A

We agree that the T210K mutant is of interest for understanding MX2 NTD-CA interactions. However, since HIV-1$_{T210K\ CA}$ is modestly susceptible to MX2 in HT0180 cells and is susceptible to MX2 in CsA treated (or CypA knock-out/mutant) HeLa or HT080s (S4 Fig and PMID: 30084827), we did not feel it was of particular interest for these investigations, as there is CypA-independent restriction of this mutant by wild-type MX2.

16. Would the NUPs requirement for MX2 in CypA KO or D66N mutant cells be similar to that of T151A?

We agree that these are interesting questions…. In our previously published work (PMID: 30084827), we investigated the effects of Nup knock-down on HIV-1 infection and MX2 activity in CsA treated cells. We would certainly be interested in repeating these experiments in CypA mutant cells with wild-type and T151A mutant MX2 but feel that it is beyond the scope of this investigation.

Reviewer #2: Line 163: the authors claim that inhibition of other primate lentiviruses by MX2 is independent of CA-CypA interactions. Since not all other primate lentiviruses were tested, the authors should specify which ones are representing such statement.

We have made this correction (Line 175)

-Usage of HIV-1NL4-3 for WT G89V A92E and G94D and NHGCapNM for N57S, N57D, N74D, G208R and T210K should be justified. What other differences are there in the sequences between NL4-3 and NHGCapNM, if they exist what could be a confounding factor in the results?

We apologize, there was an error in the initial Methods section. The viruses with NHGCapNM are actually NHGCapNM with the NL4-3 CA (NM= NotI MluI restriction sites), not the NHG capsid. We have not observed any differences between NL4-3 and NHGCapNM with NL4-3 CA (or NHGCapNM with the NHG CA) with regards to MX2 sensitivity or sensitivity to Nup depletion in multiple cell lines. We have corrected the Methods section to correct this error (Line 536)

-TRIM5alpha restriction upon blocking CypA interaction to the capsid is mentioned in the introduction but not discussed as a possible factor into many of the shown phenotypes. How do the authors see that Mx2 presence removed TRIM5alpha restriction by the proteosome (+CsA)?

We have added discussion regarding the possible role of TRIM5α in the phenotypes reported here (Lines 416-424)

Improvement of figures:

-There is quite a lot of data, and it is an impressive body of work. However, the authors should consider another representation (in parallel of as supplementary) that would be easier to read the restriction/enhancement such as ratios, log2FC or alike.

We have added figures showing log2FC to specifically show the effects of MX2 as requested (new S5, S7, S9, S11, S13, and S15). Although this adds a fair amount to the supplemental figures, we agree with the reviewer that these figures allow for improved visualization of restriction/enhancement. We considered replacing the

main figures with log2FC, but decided against this approach since that prevents visualization of differences in infectivity that are independent of MX2 (eg. a reduction in infection upon CsA treatment)

-Some graphs compare 0.1 to 100, other 0.001 to 10 IU/pg RT, many of the restriction in the higher IU/pg RT look smaller than they are, for example in Line 136 there's a "slight enhancement of infection" by G89V that appears minimal as the scale compressed the important information. For example Fig S4 N74D Ctrl data 2x~5x ratios have minimal differences in the graph and are hard to interpret.
Since the IU/pg of RT graphs are on a log-scale, the effect sizes are comparable independently of the range (eg. a two-fold change from 10 to 5 is the same distance on the graph as a two-fold change from 100 to 50). We assert that showing four logs for each virus, with the range dependent on the infectivity of a given virus is appropriate for comparing viruses of different titer (and would be similar if IU/ml were shown on a log scale). In either case, the new figures showing log2FC also resolve this issue by presenting the restriction/enhancement for each virus on the same normalized scale.

-Fig 2/3 color codes appear to be "by motif", please make it clear in the describing text or as a figure legend.
Done

-Fig 5. 11RRR15 is missing in the figure (above green T151).
Corrected

-Fig 6 and Fig S9 should have WT in legend for clarity.
Done

-Some Circles in Fig 7 only show one circle (-Dox), certainly is because they overlap, but the authors should find a way to make it clear (slight shift in the horizontal axis for example). An example is NUP153KD in HIV-1WT-MX2.
Done

-The statistical data is provided in supplemental data. Some of it should be clearly reported in the figures themselves (at least the ones described in results/discussion).
We decided to exclude statistical annotation from the figures because we were unable to determine a scientific rationale for the comparisons to include/exclude. Although the inclusion of statistical analysis for phenotypes we find of particular interest might be somewhat helpful, we are concerned that this might bias interpretation of figures by readers by eliminating other statistical comparisons.

Text improvement comments:
Line 67: "alters interactions" should be specific as to what is the phenotype of the phosphorylation of Serins.
We have clarified that phosphorylation reduces interactions with CA
Line 69-70: be more specific (at least exemplify) on what are the phenotypes to alterations of residues throughout all domains of MX2.
We have added more detail here to exemplify the altered phenotype, but have not included specific residues/phenotypes since this report is focused on GTPase activity rather than phosphorylation.
Line 79-80: regarding enzymatic activity of CypA need references.
Added
Line 109: The results section needs improvement on the experimental details (that are provided in methods) on whether TRIMCyp proteins are overexpressed/KI and that the TRIM-huCypA carry the TRIM "body" of owlTRIM and not human TRIM5alpha.
We have clarified that these experiments involve ectopic expression. The text notes that the Cyp in omkTRIMCyp is replaced with human CypA (Lines 112-114).
Line 133: Csa/HT1080 phenotype described should have a citation in this section of the text.
We may have missed something here, but there was a citation for this statement, reference #30, now on line 137.
Line 142: It is not clear if "this mutant" is relative to N57D or N57A.
We have corrected this to clarify (Line 150).
Line 191: Please specify which MX2 mutants the authors are referring to.

Done (Liens 212-214)

Line 247: Please clarify in the text to which the mutations positions "belong" to, either Mx1 or Mx2. For example K131A (K83A) give no information to what protein each belongs to respectively.

Done (Lines 291-294)
